# Psychosocial Pre-Transplant Screening With the Transplant Evaluation Rating Scale Contributes to Prediction of Survival After Hematopoietic Stem Cell Transplantation

**DOI:** 10.3389/fpsyt.2021.741438

**Published:** 2021-10-08

**Authors:** Simon Scherer, Christoph Scheid, Michael von Bergwelt, Martin Hellmich, Christian Albus, Frank Vitinius

**Affiliations:** ^1^Department of Psychosomatics and Psychotherapy, Faculty of Medicine and University Hospital Cologne, University of Cologne, Cologne, Germany; ^2^Department of Pediatric Surgery and Pediatric Urology, University Children's Hospital, University Hospital Tuebingen, Tuebingen, Germany; ^3^Department I of Internal Medicine, University of Cologne, Faculty of Medicine and University Hospital Cologne, Cologne, Germany; ^4^Department III of Internal Medicine, Ludwig Maximilian University of Munich, Munich, Germany; ^5^Faculty of Medicine and University Hospital Cologne, Institute of Medical Statistics and Computational Biology, University of Cologne, Cologne, Germany

**Keywords:** pre-transplant evaluation, transplant evaluation rating scale, adherence, patient survival, graft-vs.-host disease, medication experience scale for immunosuppressants

## Abstract

There is no standard in hematopoietic stem cell transplantations (HSCT) for pre-transplant screening of psychosocial risk factors, e.g., regarding immunosuppressant non-adherence. The aim of this prospective study is to explore the predictive value of the pretransplant psychosocial screening instrument Transplant Evaluation Rating Scale (TERS) for mortality in a 3-year follow-up. Between 2012 and 2017 61 patients were included and classified as low (TERS = 26.5–29) and increased-risk group (TERS = 29.5–79.5). Both groups were compared regarding mortality until 36 months after transplantation and secondary outcomes [Medication Experience Scale for Immunosuppressants (MESI); incidence/grade of GvHD]. The increased-risk group (*n* = 28) showed significantly worse cumulative survival in the outpatient setting (from 3 months to 3 years after HSCT) [Log Rank (Mantel Cox) *P* = 0.029] compared to low-risk group (*n* = 29) but there was no significant result for the interval immediately after HSCT until 3 years afterwards. Pre-transplant screening with TERS contributes to prediction of survival after HSCT. The reason remains unclear, since TERS did not correlate with GvHD or MESI. The negative result regarding the interval immediately after HSCT until 3 years could be caused by the intensive in-patient setting with mortality which is explained rather by biological reasons than by non-adherence.

## Introduction

The number of patients receiving hematopoietic stem cell transplantation (HSCT) in Germany has exceeded 3,000 annually since 2011, with the highest number of 3,506 recorded in 2018 (1). The number of HSCTs around the world increases each year, with 22,000 recently recorded in the US and around 40,000 in Europe (2, 3). Patients receiving stem cell transplantation must cope with post-treatment consequences: they can suffer from life-threatening or potentially lethal complications, as well as from severe psychological strain (4). Risk stratification is increasingly coming into focus, as early identification of barriers may help to prevent deadly events. Responsible for the majority of complications that follow HSCT is the graft-vs.-host disease (GvHD) (5, 6). Adherence to medication during and after transplantation plays a key role in survival after HSCT and non-adherence to medication is directly associated with GvHD (7–11). In a recent study, adherence was measured 60–180 days post-transplant using a self-administered questionnaire, which showed that 54.6% of patients were poorly adherent (12). Prior studies have examined the relationship between adherence, psychosocial variables, and outcome after both solid organ and stem cell transplantation (13–15). Psychosocial factors, such as prior psychiatric history or poor coping skills, have a significant impact on survival after HSCT (13). Screening tools, such as the Transplant Evaluation Rating Scale (TERS), have been introduced and used to evaluate the psychosocial aspects of HSCT in advance (16–18). A pre-transplant survey has been shown to predict psychosocial outcomes 12 months after transplantation, while others have shown that higher psychosocial strain correlates with higher readmission rates (13, 15). A previous retrospective study showed that lower TERS scores could predict a benefit in overall survival in a 1-year follow-up; further, it was found that HSCT recipients had lower readmission rates 90 days after transplantation (13, 15). A prospective study noted a survival advantage after HSCT, for low/moderate-risk patients compared with high-risk patients at day 100, 1 year and overall with a median follow-up of 48 months (19). In addition, TERS has been validated specifically for HSCT patients and has shown a high interrater reliability, which makes it useful for standardization and comparison (16, 20, 21).

The main objective of the present prospective study is to analyse whether TERS can contribute to prediction of overall survival after HSCT and validating pre-HSCT using an assessment of adherence by physicians. It is hypothesized that pre-transplant identification of psychosocial strain can predict survival after HSCT. The second objective is the assessment of patients attitudes to immunosuppressive therapy after organ transplant by using the Medication Experience Scale for Immunosuppressants (MESI) as a proxy parameter for adherence, which may influence adherence to therapy after HSCT (22). To verify the hypotheses, TERS and MESI screenings were utilized in combination with GvHD gradings in a three-year follow-up. To our knowledge, this is the first prospective long-term study to comprehensively address psychological variables, survival data and barriers of adherence in patients before and after HSCT.

## Materials and Methods

**Figure 1:**
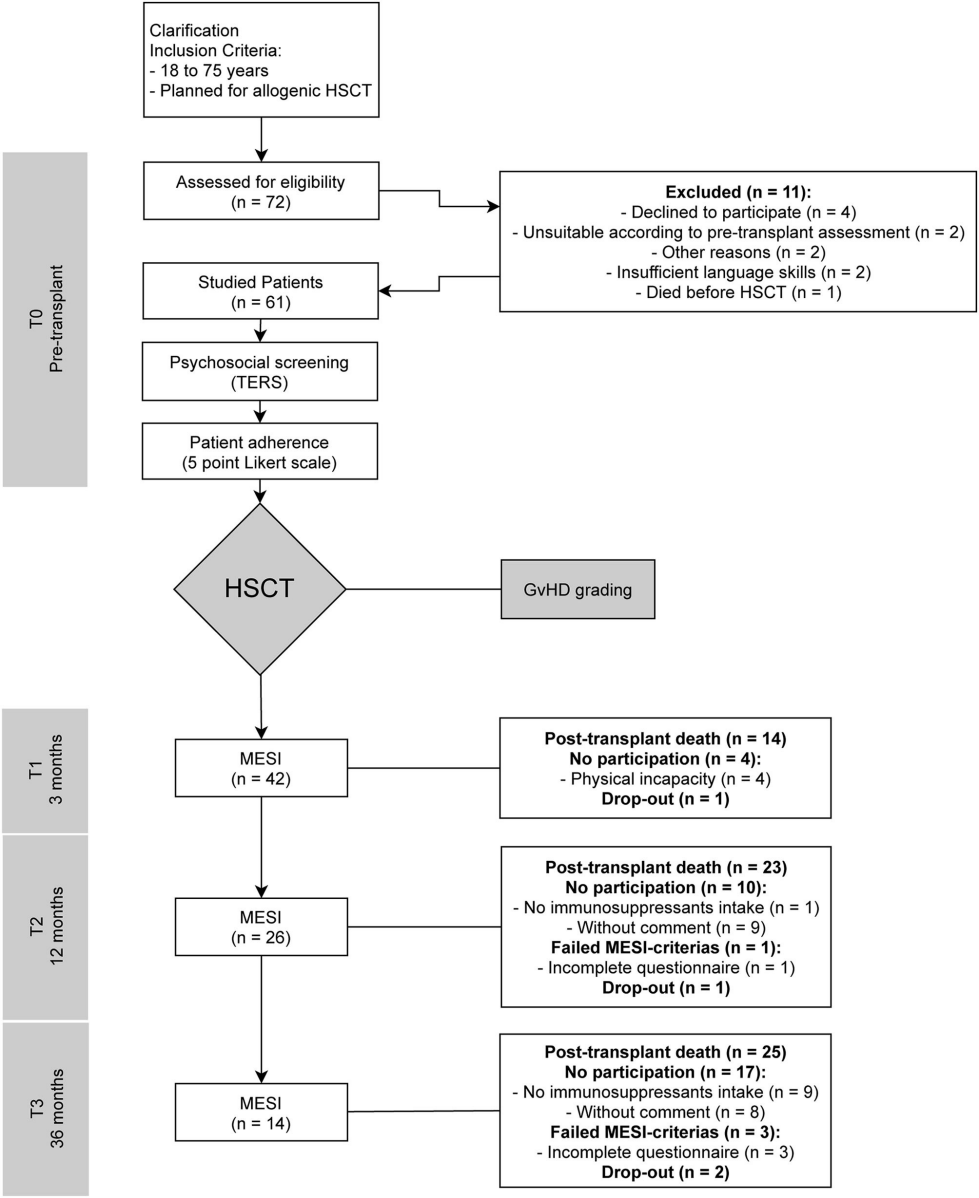
Trial flow-chart. T0, pre-transplant; T1, three months post-transplant; T2, twelve months post-transplant; T3, 36 months post-transplant; HSCT, hematopoietic stem cell transplantations; TERS, Transplant Evaluation Rating Scale; SIRT, Structured Interview for Renal Transplantation; GvHD, graft-vs.-host disease; MESI, Medication Experience Scale for Immunosuppressants.

A prospective study was conducted, and data were collected from listed patients for allogeneic bone marrow transplantation in the Department I of Internal Medicine of the University Hospital of Cologne at four specific time points for each individual: before transplantation (T0) and three (T1), twelve (T2), and 36 months (T3) after HSCT. The study was conducted with approval by the ethics committee of the University of Cologne (21^st^ May 2012 and 12^th^ September 2016, Code: 09-175) in accordance with the principles of the World Medical Association's Declaration of Helsinki (2008). Written informed consent was obtained prior to participants' inclusion in the study. The study was registered in the German Register for Clinical Trials (DRKS) DRKS-ID: DRKS00011762. The HSCTs took place between August 2012 and November 2014. Figure 1 presents the trial profile: A first assessment was conducted during the patients' preparation for the planned allogeneic transplantation (T0), including TERS, a modified version of the Structured Interview for Renal Transplantation (SIRT) adapted for HSCT and a five point Likert scale in which attending physicians were asked to rate the individual patient adherence (23). Patients completed the MESI at T1, T2, and T3. Additional information was collected from medical records. The original question of whether TERS could predict adherence was changed due to lost to follow-up numbers and mortality. We thus used MESI as our adherence measure, or as a proxy parameter to represent barriers to adherence, then performed a *post-hoc* analysis regarding survival and created Kaplan-Meier curves, and extended the scope of this work. With the extended question, we refer to Vitinius and Reklat et al. where this question was addressed in relation to heart transplant patients (24).

### Transplant Evaluation Rating Scale

The TERS is an expert rating instrument that includes 10 separate psychosocial domains with variable weighting on a three-point rating scale (good, moderate, and insufficient) (16). The domains include psychiatric history with axis-I or -II disorders, substance use/abuse, compliance, health behavior, quality of family and social support, history of coping, dealing with disease and treatment, quality of affect, and mental status. Domain subtotals were added to reach a final score of between 26.5 and 79.5. In the present study, missing or ambiguous data were rated with the lowest score. The TERS ratings for all included patients were conducted by two health care professionals, the author and a study nurse.

Hoodin et al. conducted several studies on HSCT using TERS as a pre-transplant screening tool (16, 20, 25). In the present study, the classification was adapted from previous studies, with three different groups according to score: low- (26.5–29), moderate- (29.5–37), and high-risk (37.5–79.5) (17, 20). Higher scores correlate with greater impairment in psychosocial functioning (26). After data collection, an unfavorable distribution within the risk groups became apparent. We therefore decided to adapt the classification by combining the moderate- and high-risk groups into a single increased-risk group (29.5–79.5). This enabled the two low-risk (*n* = 29) and increased-risk (*n* = 28) groups, comprising a nearly identical number of patients to be compared, and thus still remain consistent with the existing classification.

### Modified Structured Interview for Renal Transplantation

The SIRT is a professionally developed tool for psychiatric interviewing performed by clinicians for renal transplant recipients (23). The main categories are background/demographic information, understanding of illness, education/socioeconomic status, brief family history, coping/personality style, psychiatric history, mental status exam, and additional information. In the present study, the SIRT was adapted for HSCT patients. Modifications were related to the wording, such as changing “renal” or “kidney” to “stem cell transplantation” and leaving out specific renal subsections (e.g., dialysis).

### Patient Adherence

Patient adherence was measured before HSCT with a maximum of five points on a Likert scale by the attending physicians. Individual scores were differentiated by the following information: The patient “1 strongly agrees” representing the best score, “2 agrees,” “3 neither agrees nor disagrees,” “4 disagrees,” and “5 strongly disagrees” meaning non-adherence.

### Medical Outcomes: Primary Disease and Graft-vs.-Host Disease Grading, Disease Risk Index, Mortality, Comorbidities and Hospitalization

Clinical parameters, such as primary disease with individual stage at T0, GvHD, the disease risk index (DRI), hospitalization, comorbidities including the Hematopoietic Cell Transplantation-Specific Comorbidity Index (HCT-CI), and mortality data, were collected from medical records and computed if necessary. We used the validated and refined DRI based on Armand et al. which is used to stratify the individual disease risk (27). The patients are divided into four groups with different overall survival: Low, intermediate, high, and very high. The HCT-CI tries to predict the probability of non-relapse mortality and survival following allogeneic HSCT and categorizes patients into three risk groups (low-, intermediate-, and high-risk) by means of score (28). At times, ratings changed during hospitalization or were defined with decimals. In this case, the overall highest score was used after being rounded up. Data regarding mortality, which were regularly updated until November 2017, were collected through the hospital information system.

### Medication Experience Scale for Immunosuppressants

The MESI is a psychosocial screening tool used to evaluate the subjective experience and attitude of patients following organ transplant. It consists of seven items and registers the patient's individual experience of their immunosuppressive medication. It shows an internal consistency (Cronbach's alpha) of 0.78. Items 1–3 deal with side effects, while the remainder of the items deals with dosing, duration of effect, “estimation to be harmed,” and agreeableness. The cut-off value is 15 (range 4–33 points), and a score higher than 15 indicates a significantly increased risk of “limited” compliance (22).

### Statistical analysis

The qualitative variables were summarized by count (percentage) and the quantitative variables by mean ± standard deviation or median (range), contingent on distributional characteristics (e.g., skewness).

Distributions of time-to-event data were summarized by Kaplan-Meier curves and were compared with the log-rank test. Moreover, multiple regression models (logistic or Cox) were used to further explain correlations in relation to adherence and the variation in survival. *P*-values < 0.05 were considered statistically significant. All calculations were done with the software SPSS Statistics (IBM Corp., Armonk, NY. USA).

## Results

### Patient Characteristics

The patient characteristics and sociodemographic data are presented in Table 1. Data were collected from 61 of 72 eligible patients who underwent allogenic HSCT with details showed in Figure 1. Of the 11 patients who declined to be included in the study, five were women, three men, and the gender of three was unknown. A further two patients dropped out during the study without reported information.

**Table 1:** Patient characteristics.

		***N* (%)**	***d***	**Group mortality % (overall %)**
**Gender**				
	Female	23 (37.7%)	–	56.5 (21.3%)
	Male	38 (62.3%)	–	42.1 (26.2%)
**Age (at T0)**				
	Mean ± SD	52.6 ± 15.4	–	–
	Median (range)	57 (19–76)	–	–
	TERS low-risk group	53.86 ± 15.9	–	–
	TERS moderate-risk group	51.18 ± 15.8	–	–
	TERS high-risk group	54 ± 10	–	–
	TERS increased-risk group	51.79 ± 14.63	–	–
**Overall mortality**				
	Survived	32 (52.46%)	–	–
	Died	29 (47.54%)	–	–
**Died post-transplant after (days)**				
	Mean ± SD	–	869.38 ± 635.56	–
	Median (range)	–	1 096.5 (14–1,729)	–
**School education**				
	High school	39^*^ (69.6%)	–	48.7 (31.2%)
	Less than high school	17 (30.4%)	–	35.3 (9.8%)
**Family status**				
	Single or unknown	15 (24.6%)	–	40 (9.8%)
	In a relationship	46 (75.4%)	–	47.83 (36.1%)
**Primary disease**				
	Acute myeloid leukemia	28 (45.9%)	–	50 (23%)
	B-cell-lymphoma	13 (21.3%)	–	53.85 (11.5%)
	Acute lymphoblastic leukemia	5 (8.2%)	–	80 (6.6%)
	T-cell-lymphoma	5 (8.2%)	–	20 (1.6%)
	Myeloproliferative neoplasm	5 (8.2%)	–	60 (4.9%)
	Aplastic anemia	2 (3.3%)	–	–
	Myelodysplastic syndrome	2 (3.3%)	-	–
	Richter's transformation	1 (1.6%)	–	–
**Patient details**				
Comorbidities (HSCT-CI Score)	Overall, Mean ± SD	2 ± 1.9	–	–
	TERS low-risk group, Mean ± SD	3 ± 1.7	–	–
	TERS moderate-risk group, Mean ± SD	2 ± 1.7	–	–
	TERS high-risk group, Mean ± SD	2 ± 1.7	–	–
	TERS increased-risk group, Mean ± SD	2 ± 1.9	–	–
Disease risk index	Low	6 (9.8%)	–	33.3 (9.8%)
	Intermediate	35 (57.4%)	–	42.9 (24.6%)
	High	13 (21.3%)	–	53.9 (11.5%)
	Very high	7 (11.5%)	–	71.4 (8.2%)
**Hospitalization**				
	Overall, Mean ± SD	–	42.62 ± 12.46	–
	TERS low-risk group, Mean ± SD	–	43.87 ± 11.74	–
	TERS moderate-risk group, Mean ± SD	–	40.56 ± 10	–
	TERS high-risk group, Mean ± SD	–	34 ± 8.63	–
	TERS increased-risk group, Mean ± SD	–	39.13 ± 9.95	–
	Overall, Median (range)	–	41 (21–91)	–
	TERS low-risk group, Median (range)	–	42 (32–91)	–
	TERS moderate-risk group, Median (range)	–	39.5 (29–72)	–
	TERS high-risk group, Median (range)	–	34 (21–44)	–
	TERS increased-risk group, Median (range)	–	39 (21–72)	–

* *Unknown information (n = 5)*.

### Patient Adherence

Mean adherence score of all 61 patients evaluated by the attending physicians, was 2. Patient adherence correlates significantly with the TERS (T0) scores (*P* = 0.011, *r* = 0.36).

### Survival

**Figure 2:**
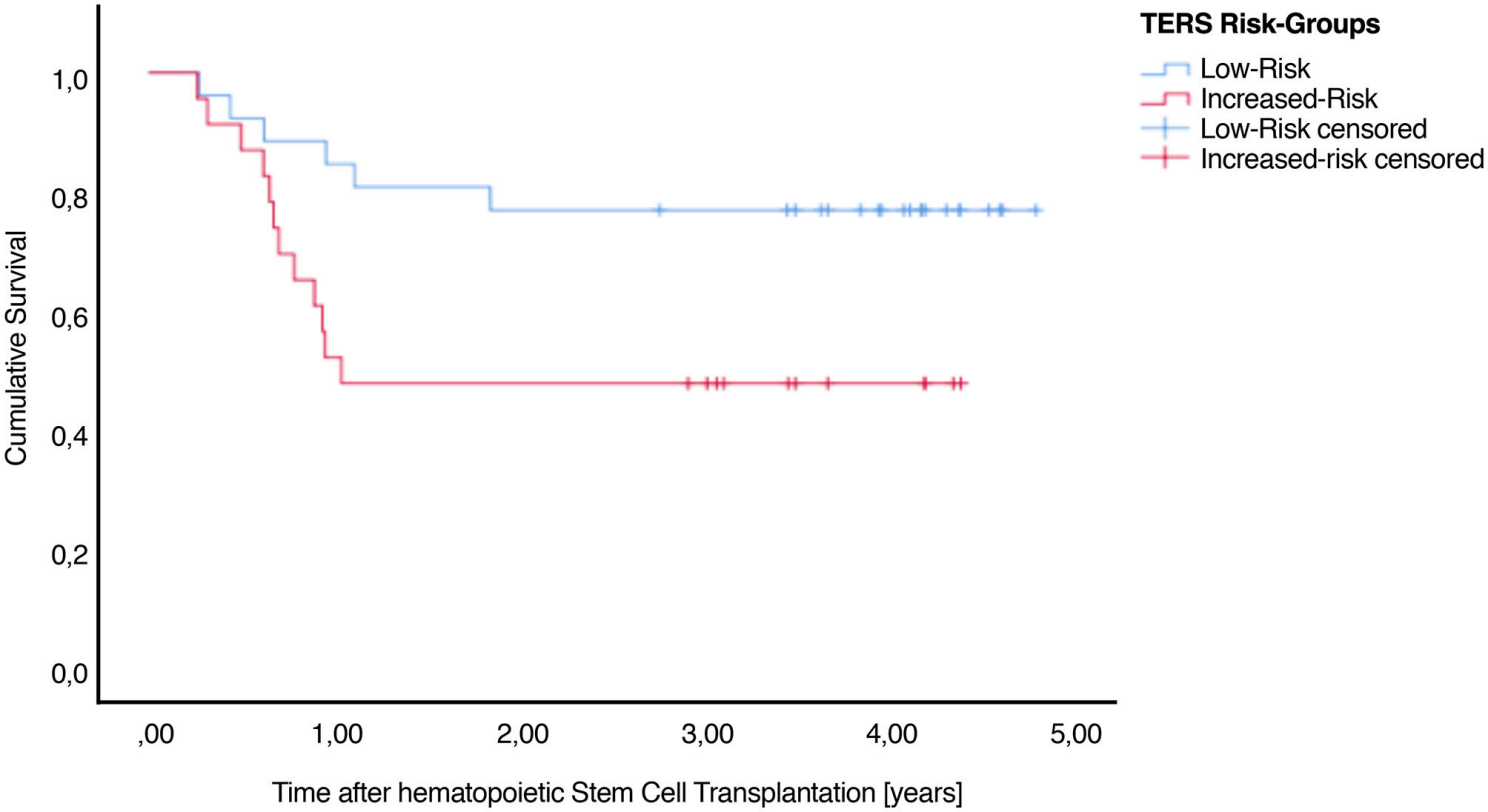
Cumulative survival after HSCT illustrating the association between TERS psychosocial risk scores. There is a significant difference between low-risk and increased-risk patients.

A total of 33 patients (53.23%) were still alive at the end of our evaluation. Higher TERS scores were correlated with lower survival (Figure 2). Table 2 presents the TERS scores and mean survival, and outpatient settings are highlighted, specifically 90 days after HSCT. An estimated survival rate of 75.9% was observed for low-risk patients (lower bound 60.3%, upper bound 91.4%) and an estimated survival rate of 44.4% for increased-risk patients (lower bound 25.7%, upper bound 63.2%) for 90 days post-HSCT survivors. Here, the hazard ratio was high, with 2.399 (95% CI 1.055–5.444). Including all days after HSCT, a non-significant result (*P* = 0.09) was observed. The mean survival time was 2.78 (SE 0.28) years for all patients. The mean survival time of low-risk patients was 3.84 (SE 0.32) years and for increased-risk patients was 2.43 (SE 0.38) years [Log Rank (Mantel Cox) *P* = 0.029] during our total measurement period of 5 years in outpatient setting.

**Table 2:** Transplant evaluation rating scale (TERS) results.

**TERS total score**	***n***					
Mean ± SD	31.1 ± 6.7					
Median (range)	29 (26.5–57.5)					
**TERS risk groups**	***n***	**Mortality (** ***n*** **)**	**Mean survival (years)**	***P*** **-value** ^**b**^	**Mean survival, outpatient (years)** ^**a**^	***P*** **-value** ^**b**^
Low-risk	31 (52.54 %)	9 (35.48 %)	3.25 ± 0.36		3.84 ± 0.32	
Moderate-risk	22 (37.29 %)	14 (63.64 %)	1.95 ± 0.39		2.34 ± 0.42	
High-risk	6 (10.17 %)	3 (50 %)	2.32 ± 0.71	0.163	2.32 ± 0.71	0.085
Increased-risk	28 (47.46 %)	17 (60.71 %)	2.1 ± 0.36	0.09	2.43 ± 0.38	0.029

a *Outpatient Setting, survived >90 days*.

b *Log Rank (Mantel-Cox)*.

### Graft-vs.-Host Disease

The results of the present study regarding GvHD are shown in Table 3. Statistical analyses included Kaplan-Meier survival curves. We observed a statistically significant correlation in terms of maximum GvHD grade and TERS risk-groups (*P* = 0.036). Moreover, liver GvHD grade correlates regarding survival [Log Rank (Mantel Cox) *P* = 0.013] but no other significant data regarding survival and skin, intestinal or maximum GvHD grade can be seen in the sample.

**Table 3:** Graft-vs.-host disease (GvHD) results.

**GvHD manifestation**		***P*-value** ^**b**^
Yes (*n*)	50 (82%)	
No (*n*)	11 (18%)	
**GvHD mortality**		
Deceased with GvHD (*n*)	21 (65.6%)	
Deceased without GvHD (*n*)	8 (27.6%)	
Overall Survival (years)^a^	3.3 ± 0.27	
**Skin GvHD**		
*n*	44	
Mean grade ± SD	1.8 ± 1.2	0.275
TERS low-risk average grade (*n* = 22)	1.86	
TERS increased-risk average grade (*n* = 22)	2	
**Liver GvHD**		
*n*	3	
Mean grade ± SD	0.2 ± 0.7	0.013
TERS low-risk average grade (*n* = 2)	0.23	
TERS increased-risk average grade (*n* = 1)	0	
**Intestine GvHD**		
*n*	13	
Mean Grade ± SD	0.5 ± 1.1	0.464
TERS low-risk average grade (*n* = 7)	0.45	
TERS increased-risk average grade (*n* = 6)	0.29	
**GvHD sum score**		
Mean ± SD	1.5 ± 1	
**Maximium GvHD grade-survival**	(years)	
0	2.3 ± 0.6	
1	3.3 ± 0.6	
2	3.7 ± 0.5	
3	3.1 ± 0.4	
4	2.6 ± 1.4	0.753

a *Outpatient setting, survived >90 days*.

b *Log Rank (Mantel-Cox)*.

### Medication Experience Scale for Immunosuppressants

The sum scores of MESI are provided in Table 4. Our data does not show significant MESI vs. TERS correlation in inpatient (T1: *P* = 0.85, *r* = −0.026; T2: *P* = 0.12, *r* = −0.206) or outpatient setting (T1: *P* = 0.96, *r* = −0.009; T2: *P* = 0.9, *r* = 0.03). All survivors were contacted at specific time points and were reminded to answer the questionnaire, if necessary. Forty-two patients completed the questionnaire 3 months after bone marrow transplantation (T1). Four patients were physically unable to complete the test at that time point, and one patient dropped out. Twenty-six patients filled out the MESI again 1 year after transplantation (T2), while one patient did not fill out the questionnaire completely, which resulted in a lower overall score. Ten were unable to hand in the test: nine patients did not provide any comment, and one patient was no longer taking immunosuppressants. Fourteen patients completed the MESI 3 years after HSCT (T3). A total of 17 did not fill MESI: nine commented that they were not taking immunosuppressants and, therefore, did not answer the questionnaire. The other eight did not leave any comment. An additional drop-out also reduced the number of participants.

**Table 4:** Medication experience scale for immunosuppressants (MESI) results.

	**MESI T1 (*n* = 42) sum score**	**MESI T2 (*n* = 26) sum score**	**MESI T3 (*n* = 14) sum score**
Mean ± SD	16.5 ± 2.2	17.7 ± 3.1	14.9 ± 6.2
Median (range)	16 (14–22)	17 (14–25)	15 (5–28)
	**MESI T1 (*****n*** **=** **42) sum score (*****n*****)**	**MESI T2 (*****n*** **=** **26) sum score (*****n*****)**	**MESI T3 (*****n*** **=** **14) sum score (*****n*****)**
Adherence	17 (40.5 %)	14 (53.9 %)	7 (50 %)
Limited adherence	25 (59.5 %)	12 (46.2 %)	7 (50 %)
TERS low-risk group, limited adherence	16 (64%)	9 (75%)	5 (71.4%)
TERS moderate-risk group, limited adherence	8 (32%)	3 (25%)	1 (14.3%)
TERS high-risk group, limited adherence	1 (4%)	–	1 (14.3%)
TERS increased-risk group, limited adherence	9 (36%)	3 (25%)	2 (28.6%)

*T0, pre-transplant; T1, three months post-transplant; T2, twelve months post-transplant; T3, 36 months post-transplant; Limited adherence, MESI sum-score >15*.

## Discussion

To our knowledge, this is the first prospective 3-year follow-up study to demonstrate significant differences in survival after HSCT assessed by pre-transplant evaluation using TERS low- and increased-risk groups in the outpatient setting. Increased-risk patients (*n* = 28) showed a higher mortality compared with low-risk patients (*n* = 31). Our results also provide evidence, that pre-transplant (T0) TERS scores correlate significantly with adherence. Kaplan-Meier curves were calculated for both inpatient and outpatient settings, and significant results were noted for the outpatient setting only. This could be due to the intensive inpatient setting, which offers sufficient support for medication adherence, and by mortality that occurred due to biological reasons rather than due to non-adherence.

Describing and analyzing risks in advance should be an important element in modern HSCT therapy. Prior studies with different approaches have demonstrated an association between psychosocial pre-transplant risk factors and therapy success in HSCT patients, affecting length of hospitalization, readmission rates and overall survival (13, 19, 25, 29–35). Hoodin et al. used TERS multiple times, in a 5-year follow-up study as well as in a 2003 study, but TERS scores were assigned retrospectively (16, 20). Murphy et al. did not use TERS in 1996, but instead used the Composite International Diagnostic Interview to evaluate HSCT outcomes in a mean follow-up time of 82.1 months (33). Speckhart et al. described data in a poster in 2006 and showed that TERS scores correlated with the length of hospitalization (31). In their prospective approach, primarily after autologous transplant initiated in an outpatient setting, the follow-up time was not mentioned. As such, the data from the present study is not comparable. Speckhart et al. described a significant survival advantage for low/moderate-risk TERS risk groups in a prospective 2-year follow-up study in 2014 (19). However, only an abstract that mentions a few details is available. Schumacher et al. utilized a different approach, employing the European Organization for Research and Treatment of Cancer Quality of Life Questionnaire (EORTC QLQ-C30), Resilience Scale (RS-25), and Hospital Anxiety and Depression Scale (HADS) in a prospective study with a 12-month follow-up (29). The authors measured significant changes in quality of life and depression, but not in a pathological range. Their information was not used to determine whether there was an impact on survival. In contrast, other studies have neglected to identify psychological variables that can affect post-HSCT outcomes (30, 33, 35).

Based on the above-mentioned studies that assessed pre-transplant evaluation, TERS was chosen in the present study because it has previously been successfully employed with a focus on transplantation and aim of predicting survival. A significant difference in predicting length of hospitalization, as well as 100-day, 1-, and 2-year overall survival and readmission rates could be detected between the different TERS risk-groups for HSCT patients (13, 19, 31). The results of the present study support the efficacy of pre-HSCT psychosocial screening with TERS after a 3-year follow-up, identifying a correlation between significantly worse survival with patients classified in the increased-risk group.

Psychosocial strain is associated with poor adherence to medical treatment (36–38), correlates negatively with clinical recovery and survival (37), and impacts on emotional distress, quality of life, and overall health outcomes (36). It has been shown that non-adherence during and after therapy may result in reduced clinical outcomes and overall survival in a <2-year median observation period (8, 39, 40). Moreover, adherence itself may be negatively affected by psychosocial risk factors and may influence overall survival (25, 41, 42). In the present analysis, significantly worse cumulative survival was noted in the outpatient setting (from 3 months to 3 years after HSCT). Including inpatient stays in the present calculations yielded no significant results. This could be explained by biological reasons rather than by non-adherence because patient care on bone marrow and stem-cell transplantation wards is highly intensive and includes support of immunosuppressant adherence. Indeed, HSCT recipients show the highest rates of non-adherence out of all patients with cancer, which increases the potential of life-threatening complications (43, 44), while research has shown decreasing adherence over time in outpatient regimens (8). Other factors that may influence survival after HSCT such as concomitant diseases are part of current discussions. The HCT-CI was established and partially validated to summarize possible secondary factors. Matching the TERS risk stratification, patients are divided into risk groups with HCT-CI score of 0 (low-risk), 1–2 (intermediate-risk), and ≥3 (high-risk) (45). But there is still disagreement about the quality of its predictive potential as e.g., Birninger et al. presented for high-risk AML patients (46). The underlying diseases with individual stages, since stem cell transplantation is performed for the therapy of various malignant and non-malignant diseases, are other biological reasons that may affect prognosis and survival (47, 48). Since oncological patients have a doubled suicide rate compared to the general population, this fact could also increase mortality in our patient cohort (49).

A further aim of the present study was to identify potential psychological risk factors for HSCT recipients to optimize therapy and improve survival rates. In addition, the present research aimed to validate the capacity of the TERS to predict survival in HSCT patients in a comprehensive study. Based on TERS screening, patients at risk could be identified upfront and treated with targeted therapies to help improve survival. The present study suggests that psychosocial strain in general has an impact on mortality. However, the specific psychosocial risk factors that are directly related to increased mortality remain unclear. It was also not possible to point out other significant statistical correlations except survival vs. liver GvHD/maximum GvHD grade, that could support existing theories. Due to the small numbers, random significance is most likely to be the case and we do not link the presence of GvHD to a higher mortality rate, as showed by e.g., Pereira, Arai, Flowers, and Gresch, etc., before. Our results still leave GvHD as an unpredictable stand-alone parameter, even though several prior studies have sought to identify predictive factors for GvHD like problematic compliance, pre-transplant liver condition, non-adherence, inter alia HLA-mismatch, irradiation, and age (7, 50–52). In the present study, only the levels of psychosocial functioning measured by TERS could help predict overall survival, which seemed otherwise unaffected by age and GvHD for example.

All patients in the present study were examined by psychologists in a routine pre-transplant screening. The psychologists assessed every individual in an open conversation for at least 30 minutes, examined psychosocial diseases, social support, and mentally prepared them for the upcoming stress during and after HSCT. Two patients in the present study were declared unsuitable for HSCT in the routine screening during the observation period. One homeless person was rejected. Another person's HSCT was postponed due to insufficient social support. The rejected patients could have had higher TERS scores. Therefore, all transplanted patients in our study were accepted by routine psychosocial pre-transplant screening. This may be a factor explaining why, compared with the results of other research, such as that by Twillman et al. (33.5) and Hoodin et al. (35.3 and 38.5), our patients (31.1) showed lower mean TERS scores (16, 17, 20). Our comparatively low TERS scores, the use of cut-off values by Hoodin et al. and the small sample size may have contributed to a smaller high-risk number of patients that showed that our routine psychosocial pre-transplant screening was successful. An agreement on cut-off values for low- or high-risk patients is overdue. Nevertheless, TERS appears to be a valuable psychosocial assessment instrument. As Vitinius and Reklat et al. discussed in relation to heart transplantation patients (24), different cut-off values exist. They showed significant differences in survival between TERS risk-groups for patients on the waiting list for heart transplantation, using Rothenhäuslers cut-off values, which are often used in solid organ transplantation, but not for Hoodin cut-off values (53).

Our data have certain limitations, such as the use of a single-center design with a relatively small sample size. However, the individual approach, whereby all questionnaires were administered by just two individuals, resulted in a low number of drop-outs beyond the high mortality in the sample. This continuity meant that a homogeneous form of questioning and survey could be guaranteed. External assessment of adherence was performed by a total of five different treating physicians. The data were sent to us over the course of the study; the exact time of collection is unclear. To minimize the burden of data collection on patients and because there is no uniform clinical follow-up for the heterogeneous underlying diseases of these patients, post-HSCT follow-up was performed by mail. Therefore, only self-report questionnaires could be used. Information regarding mortality has been obtained from the medical records only. We might otherwise have more information on, for example, causes of death. In addition, in order not to reduce the power of the study by statistical complexity, patient data, concomitant diseases, and disease stages, were listed and should be considered only as a signal. Future studies focusing on more patients with homogenic disease and other psychosocial screening tools, such as the Stanford Integrated Psychosocial Assessment for Transplant (SIPAT), could corroborate the results of the present study. The SIPAT has been used to date in solid organ transplantations only and may provide extended insight into pre-transplant psychological risk. More recently, a retrospective study showed significant correlations using SIPAT in predicting non-adherence after HSCT for patients at high psychosocial risk, leading to medical morbidity (54). Further, intervention studies are needed to confirm the TERS' clinical efficacy and help establish systematic pre-transplant screenings with TERS. In addition, concrete measurement of medication intake (e.g., by Medication Event Monitoring System (MEMS) could be included to assess adherence, rather than only barriers regarding immunosuppressive medication, as in our case with MESI. Unfortunately, the use of monitoring systems is expensive in routine care.

Investigations into the influence of extended psychosocial variables with the goal of identifying predictors for decreased survival rates, such as depression or substance abuse and poor adherence to therapy, are needed. Other parameters of interest include measurements of health issues, quality of life, and social support, as well as perceived social support, with instruments like the Patient Health Questionnaire (PHQ), Fragebogen zur Sozialen Unterstützung (F-SozU), and HADS (29, 55, 56). The present study addressed only acute GvHD, though chronic graft-vs.-host disease (cGvHD) is the leading cause of late treatment-related deaths after HSCT, and prior research has shown a correlation between medication non-adherence and GvHD manifestation (7, 57). Including pre-transplant screening and standardized cGvHD measurings could contribute to new findings. A larger collective within multi-center studies could be appropriate for comparing instruments such as the TERS and SIPAT and could validate the results of the present study on a larger scale. Together, these approaches could establish comprehensive screening tools capable of reliably identifying increased-risk patients, allowing for tailored individual pre- and post-transplant interventions to reduce negative outcomes and improve overall survival.

## Conclusion

We explored the associations between pre-transplant TERS-screening, an assessment of adherence by physicians, a MESIs, incidence/grade of GvHD, and overall survival. We found that patients with higher TERS-scores showed a significantly higher mortality in the outpatient setting. However, the reason remains unclear. A pre-transplant TERS screening can help identify patients at greater risk and offer them targeted interventions. This may contribute to improve overall survival after HSCT. Further longitudinal data with larger sample sizes could provide confirmation of our results and identify additional predictive variables for non-adherence after HSCT in order to confirm adequate pre-transplant screening instruments for identifying risk patients.

## Data Availability Statement

The raw data supporting the conclusions of this article will be made available by the authors, without undue reservation.

## Ethics Statement

The studies involving human participants were reviewed and approved by Ethics Committee of the University of Cologne (21st May 2012 and 12th September 2016, Code: 09-175). The patients/participants provided their written informed consent to participate in this study.

## Author Contributions

SS contributed to the study design, data collection, data analysis, figures, and writing. MH contributed to study design, writing, proofreading, and was responsible to data analysis. CS contributed to the study design, data collection and proofreading. MB contributed to the study design and proofreading. CA contributed to proofreading. FV contributed to the study design, data analysis, writing, and proofreading. All authors contributed to the article and approved the submitted version.

## Funding

This work was funded by German Federal Ministry of Education and Research (BMBF) grant 01KN1106.

## Conflict of Interest

The authors declare that the research was conducted in the absence of any commercial or financial relationships that could be construed as a potential conflict of interest.

## Publisher's Note

All claims expressed in this article are solely those of the authors and do not necessarily represent those of their affiliated organizations, or those of the publisher, the editors and the reviewers. Any product that may be evaluated in this article, or claim that may be made by its manufacturer, is not guaranteed or endorsed by the publisher.
